# Dynamic Prediction of Rectal Cancer Relapse and Mortality Using a Landmarking-Based Machine Learning Model: A Multicenter Retrospective Study from the Italian Society of Surgical Oncology—Colorectal Cancer Network Collaborative Group

**DOI:** 10.3390/cancers17081294

**Published:** 2025-04-11

**Authors:** Rossella Reddavid, Ugo Elmore, Jacopo Moro, Paola De Nardi, Alberto Biondi, Roberto Persiani, Leonardo Solaini, Donato P. Pafundi, Desiree Cianflocca, Diego Sasia, Marco Milone, Giulia Turri, Michela Mineccia, Francesca Pecchini, Gaetano Gallo, Daniela Rega, Simona Gili, Fabio Maiello, Andrea Barberis, Federico Costanzo, Monica Ortenzi, Andrea Divizia, Caterina Foppa, Gabriele Anania, Antonino Spinelli, Giuseppe S. Sica, Mario Guerrieri, Roberto Polastri, Francesco Bianco, Paolo Delrio, Giuseppe Sammarco, Micaela Piccoli, Alessandro Ferrero, Corrado Pedrazzani, Michele Manigrasso, Felice Borghi, Claudio Coco, Davide Cavaliere, Domenico D’Ugo, Riccardo Rosati, Danila Azzolina

**Affiliations:** 1 Division of Surgical Oncology and Digestive Surgery, Department of Oncology, San Luigi University Hospital, University of Turin, Orbassano, 10043 Turin, Italy; jacopo.moro@unito.it; 2 Department of Gastrointestinal Surgery, IRCCS San Raffaele Scientific Institute, School of Medicine, “Vita-Salute” San Raffaele University, 20132 Milan, Italy; elmore.ugo@hsr.it (U.E.); denardi.paola@hsr.it (P.D.N.); rosati.riccardo@hsr.it (R.R.); 3 Fondazione Policlinico Universitario A. Gemelli IRCCS, Università Cattolica del Sacro Cuore, 00168 Roma, Italy; biondi.alberto@gmail.com (A.B.); roberto.persiani@policlinicogemelli.it (R.P.); domenico.dugo@unicatt.it (D.D.); 4 General and Oncologic Surgery, Morgagni-Pierantoni Hospital, Ausl Romagna, 47121 Forlì, Italy; leonardosolaini@gmail.com (L.S.); cavalied@gmail.com (D.C.); 5 Fondazione Policlinico Universitario A. Gemelli IRCCS, UOC Chirurgia Generale 2, 00168 Roma, Italy; donatopaolo.pafundi@policlinicogemelli.it (D.P.P.); claudio.coco@unicatt.it (C.C.); 6 Department of Surgery, S. Croce e Carle Hospital, 12100 Cuneo, Italy; desireecianflocca@live.it (D.C.); diego.sasia@hotmail.it (D.S.); 7 Department of Clinical Medicine and Surgery, Department of Gastroenterology, Endocrinology and Endoscopic Surgery, University of Naples “Federico II”, 80138 Naples, Italy; milone.marco.md@gmail.com (M.M.); michele.manigrasso89@gmail.com (M.M.); 8 Division of General and Hepatobiliary Surgery, Department of Surgical Sciences, Dentistry, Gynecology and Pediatrics, University of Verona, 37129 Verona, Italy; giulia.turri89@gmail.com (G.T.); corrado.pedrazzani@gmail.com (C.P.); 9 Department of General and Oncological Surgery, “Umberto I” Mauriziano Hospital, 10128 Turin, Italy; mmineccia@mauriziano.it (M.M.); aferrero@mauriziano.it (A.F.); 10 Unita’ Operativa di Chirurgia Generale, D’Urgenza e Nuove Tecnologie, Ospedale Civile S. Agostino-Estense, Azienda Ospedaliero Universitaria di Modena, 41125 Modena, Italy; francescapecc@gmail.com (F.P.); piccoli.micaela@aou.mo.it (M.P.); 11 Department of Surgery, Sapienza University of Rome, 00185 Roma, Italy; gaetanogallo1988@gmail.com; 12 Colorectal surgical Oncology, Abdominal Oncology Department, Fondazione Giovanni Pascale IRCCS, 80131 Naples, Italy; daniela.rega@gmail.com (D.R.); p.delrio@istitutotumori.na.it (P.D.); 13 General Surgery Unit, San Leonardo Hospital, ASL-NA3sud, Castellammare di Stabbia, 80053 Naples, Italy; simogili@tin.it (S.G.); bianco.bar@tin.it (F.B.); 14 General Surgery Unit, Department of Surgery, Hospital of Biella, 13875 Biella, Italy; moibaf@gmail.com (F.M.); roberto.polastri@aslbi.piemonte.it (R.P.); 15 Chirurgia Generale ed Epatobiliopancreatica, E.O. Ospedali Galliera, 16128 Genova, Italy; andrea.barberis@galliera.it (A.B.); federico.costanzo@galliera.it (F.C.); 16 Clinica Chirurgica Universita’ Politecnica delle Marche, Ospedali Riuniti, 60121 Ancona, Italy; monica.ortenzi@gmail.com (M.O.); guerrieri.m@libero.it (M.G.); 17 Minimally Invasive and Gastrointestinal Surgery Unit, Università e Policlinico Tor Vergata, 00133 Roma, Italy; andreadivizia@gmail.com (A.D.); giuseppe.sica@uniroma2.it (G.S.S.); 18 Department of Biomedical Sciences, Humanitas University, Via Rita Levi Montalcini 4, Pieve Emanuele, 20072 Milan, Italy; caterina.foppa@hunimed.eu (C.F.); antonino.spinelli@hunimed.eu (A.S.); 19 IRCCS Humanitas Research Hospital, Via Manzoni 56, Rozzano, 20089 Milan, Italy; 20 Department of Surgical Morphology and Experimental Medicine, AOU Ferrara, 44124 Ferrara, Italy; ang@unife.it; 21 Department of Health Sciences, University of Catanzaro, 88100 Catanzaro, Italy; sammarco@unicz.it; 22 Oncologic Surgery Unit, Candiolo Cancer Institute, FPO-IRCCS, 10060 Turin, Italy; felice.borghi@ircc.it; 23 Department of Environmental and Preventive Sciences, University of Ferrara, Via Fossato di Mortara 64B, 44100 Ferrara, Italy; danila.azzolina@unife.it

**Keywords:** rectal cancer, machine learning algorithm, relapse, metastasis, recurrence, landmark analysis, competing events

## Abstract

**Background**: Almost 30% of patients with rectal cancer (RC) who submit to comprehensive treatment experience relapse. Surveillance plays a leading role in early detection. The landmark approach provides a more flexible and dynamic framework for survival prediction. **Objective**: This large retrospective study aims to develop a machine learning algorithm to profile the patient prognosis, especially the risk and the onset of RC relapse after curative resection. **Methods**: A cohort of 2450 RC patients were analyzed using landmark analysis. Model A applied a classical cause-specific Cox approach with a landmarking approach, while Model B implemented a landmarking-based RSF (random survival forest) competing risk algorithm. The two models were compared in terms of predictive and interpretative ability. A bootstrapped validation strategy was employed to validate the model’s performance and prevent overfitting. The best-performing hyperparameters were selected systematically, ensuring the model’s robustness within the landmark approach. The study assessed these factors’ importance and interactions using RSF and compared the predictive accuracy to that of the classical Cox model. **Results**: Model B outperformed Model A (mean C-index 0.95 vs. 0.78), capturing complex interactions and providing dynamic, individualized relapse predictions. Clinical factors influencing survival outcomes were identified across time with the landmark approach allowing for more accurate and timely predictions. **Conclusions**: The landmark approach offers an improvement over traditional methods in survival analysis. By accommodating time-dependent variables and the evolving nature of patient data, this approach provides a precise tool for profiling RC survival, thereby supporting more informed and dynamic clinical decision-making.

## Simple Summary

Rectal cancer relapse after curative surgery usually results in poorer survival and quality of life, so early identification of patients at high risk of relapse from rectal tumors is crucial. It is essential to offer risk-based recommendations that address the unique needs of survivors and caregivers while reducing the impact of provider shortages and managing costs for healthcare systems, survivors, and families. This large retrospective study aims to develop a machine learning algorithm using data from the RALAR study for profiling the risk and the onset of rectal cancer relapse after curative resection. Specifically, we proposed a machine learning algorithm that could assist clinicians in predicting patient prognosis by minimizing late relapse diagnosis, consequent delayed treatment, and the inefficient use of economic resources.

## 1. Introduction

Rectal cancer (RC) ranks eighth globally, with 729,833 new cases and 343,817 deaths in 2022 [1]. In Italy, in 2024, it is estimated that about 48,706 residents will have experienced colorectal cancer (CRC), with 24,200 cancer-related deaths in 2022 [2]. One-third of new CRC cases are rectal cancer cases.

Patient survival is mainly determined by stage at diagnosis; for stage I, the 5-year survival rate is superior to 90%, decreasing to 70–85% in stage II, 25–80% in stage III, and less than 10% in stage IV [3].

However, almost 30% of patients with locally advanced RC submitted to comprehensive treatment experience relapse (local recurrence and/or distant metastasis) within the first 5 years, with most of them diagnosed before the end of the third year [4]. Due to the high risk of relapse, surveillance plays a crucial role in the early detection and prompt management of RC recurrence.

No consensus has been reached on which type of exams and timing of the testing must be used during surveillance after RC treatment [5].

Typically, postoperative follow-up involves a clinical examination, routine blood tests with tumor markers, and imaging studies. Most global guidelines recommend visiting patients every 3 months for 2 years, every 6 months for 3–5 years, and then once a year [6,7,8].

Many studies, both randomized controlled trials (RCTs) and observational studies, investigated different types of surveillance to identify the best schedule regarding survival, recurrence detection rate, and the percentage of curative surgery in case of relapse. Three types of surveillance have been proposed and analyzed, including intensive, less intensive, and no follow-up, but no consensus has been found [9].

Furthermore, survival rates are typically static and calculated from the surgery date, without considering the importance of the time elapsed since surgery [10,11]. In this last decade, several authors demonstrated that the risk of death or relapse is related to time after surgery [12,13]. Therefore, the mere evaluation of oncological outcomes at baseline is not enough for long survival in terms of predicting prognosis, resulting in frequent surveillance monitoring and an increased psychological burden on patients. Conditional survival (CS) was recently introduced to provide a personalized prognosis based on the probability of surviving a certain number of years after diagnosis or treatment based on the time the patient has already survived.

However, most survival models fail to account for the time-varying effects of patient disease and procedure-related factors on survival and recurrence risk during follow-up. Landmark analysis addresses this by generating multiple datasets, each capturing the patient population at a specific time. These landmark datasets are then combined into a single “super dataset,” which can be analyzed using multivariable regression [14,15,16].

In this last decade, machine learning (ML) has gained a leading role in the medical field for profiling prognosis in cancer disease. These models can handle complex data interaction and multicollinearity issues by examining structured data derived from images and genetic and electrophysiological records [17]. Several studies reported that the ML technique was successfully employed to predict cancer relapse in different types of tumors, such as colorectal, breast, and gastric [18,19,20].

Landmarking provides a more realistic and clinically meaningful interpretation of survival outcomes, particularly in oncology, where the influence of prognostic factors may evolve with disease progression and treatment response. However, no efforts are reported in the literature to combine the dynamic landmarking approach with the improved profiling abilities of ML algorithms.

Moreover, the presence of competing risks, such as death from other causes, poses a significant challenge when estimating the probability of relapse and cancer-specific mortality in RC patients. Traditional survival models like Cox regression often struggle to properly account for these competing events, potentially leading to biased risk estimates [21]. In this context, random forest (RF) algorithms for survival analysis, mainly random survival forests (RSF), have emerged as a suitable analysis due to their ability to flexibly handle non-linear relationships, high-dimensional data, and time-dependent variables, all the while accurately incorporating competing risk frameworks. Estimating cumulative incidence functions (CIFs) within a competing risk setting, RF methods provide a more dynamic risk prediction approach, enforcing clinical decision-making for personalized follow-up strategies. Despite these advantages, their application with dynamic landmarking approaches remains scarce [22].

Hence, this large retrospective study aims to develop a landmarking-based RFS competing risk algorithm using data from the RALAR study [23] to profile patient survival and the risk and onset of rectal cancer relapse after curative resection.

## 2. Materials and Methods

The characteristics of this study are reported concisely, as the details have been extensively described in the previous RALAR study [23].

### 2.1. Patient Selection and Dataset

The RALAR database was adopted, and the data were retrospectively collected, including data for patients who submitted to RC resection between January 2000 and December 2016 in 19 Italian colorectal referral centers. All information has been anonymized to prevent patients from being identifiable. Central Ethics Committee approval was obtained by the AOU San Luigi Gonzaga Human Research institutional review board (approval date 23 October 2018, protocol number 15525).

**Figure 1:**
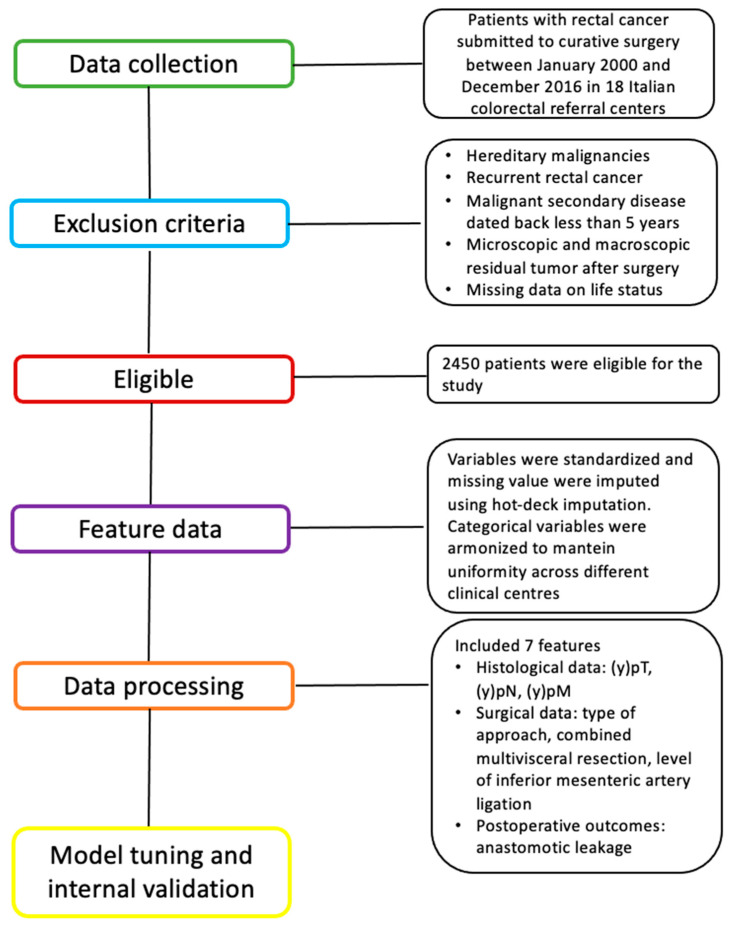
Visual diagram of the detailed process for clinical design and data collection.

Patients with hereditary malignancies, recurrent rectal cancer, malignant secondary disease dated back less than 5 years, microscopic and macroscopic residual tumors after surgery (R1 and R2), and missing data on life status were not included. After exclusion, 2450 patients were eligible for the study (Figure 1).

All patients were submitted to rectal resection and PME or TME, depending on tumor location, by open or minimally invasive (laparoscopic and robotic) approaches according to the surgeon’s preference. Based on the clinical tumor stage, patients underwent neoadjuvant therapy or upfront surgery following updated guidelines.

Adjuvant chemotherapy was administered for selected patients according to pathological tumor stage and patient condition. A multidisciplinary team evaluated every decision.

### 2.2. Statistical Analysis

This study employed a landmarking approach for survival analysis alongside traditional and machine learning-based models. Two methods have been considered for comparison.

(1) Model A: the classical landmark analysis based on the cause-specific Cox model.
(2) Model B: the proposed application of landmarking-based RFS competing risk algorithm.

The primary study outcome is cancer relapse. The proposed Model B treats the relapse as the primary outcome occurring with competing cancer death and death for other causes events.

The goal was to dynamically assess cumulative incidence probabilities over time, accounting for changes in risk factors and patient conditions at predefined time points.

#### 2.2.1. Data Preparation and Preprocessing

The dataset underwent preprocessing to ensure accuracy and consistency. Given the study’s focus on long-term survival, time-to-event variables were computed from the date of surgery to either the occurrence of an event or the last available follow-up.

In landmark analyses, separate datasets are created for each specific time point, including only patients who remain at risk. These datasets are then combined into a single dataset by stacking them together. Landmark time points were selected for one year from the time of surgery up to 15 years.

#### 2.2.2. Data Description

Data have been synthesized as median and interquartile ranges for quantitative variables and absolute and relative frequencies for categorical values. The univariable cause-specific Cox hazard ratio and *p* values have been reported to describe the effect of patients’ characteristics on the hazard of cancer death, death for other causes, and relapse.

#### 2.2.3. Landmarking Approach for Time-Varying Risk Estimation

The assumption that hazard ratios remain constant over time is a limitation of traditional survival models. To address this, a landmarking framework was implemented allowing survival estimates to be dynamically updated at multiple time points rather than relying on a single baseline prediction.

In this study, landmark analyses were conducted at one, three, five, and ten years after surgery. This allowed for the assessment of how risk factors influenced survival at different follow-up intervals.

Variables were selected according to clinical judgment. These included age, sex, comorbidities (Charlson score), Body mass index (BMI), American Society of Anesthesiologists (ASA) classification, perioperative treatments (neoadjuvant/adjuvant), smoking status, surgical approach (laparoscopic, open, robotic), inferior mesenteric artery (IMA) ligation level (high tie, low tie), anastomotic dehiscence, combined multivisceral resections (MVR), operative time, blood transfusion, tumor location (high, middle, low), distance from the anal verge, pathological T stage, pathological N stage, pathological TNM stage and tumor grade.

#### 2.2.4. Model A: Survival Analysis Using Landmark Cause-Specific Cox Models

Multivariable Cox proportional hazards models were employed to examine the effects of the variables of interest and their interactions with landmark time on cancer mortality, other causes of mortality, and relapse.

Since patients could appear multiple times in the dataset at different landmark time points, a robust [24] sandwich variance estimator was used to account for within-patient correlation due to repeated measures.

#### 2.2.5. Model B Machine Learning-Based Competing Risk Survival Analysis: Landmarking with Random Survival Forests 

While Cox models assume proportional hazards and linear relationships, these assumptions do not always hold in complex clinical datasets. A landmarking approach was also applied to RSFs to capture non-linear effects and interactions. This machine learning method builds multiple decision trees to estimate survival probabilities without requiring restrictive assumptions about hazard functions.

The schematic diagram (Figure S1, Supplement) illustrates the overall workflow of the machine learning model implemented in this study, combining an RSF algorithm with a landmarking framework to perform dynamic survival prediction in the presence of competing risks.

✓ **Input Layer—Clinical Data and Landmark Time Points**: The model starts with structured clinical data, including baseline and time-varying covariates such as tumor staging, surgical approach, perioperative complications, and comorbidities. For each predefined landmark time point, a subset of patients still at risk is selected to form a “landmark dataset.”
✓ **Landmark Dataset Construction**: Separate datasets are generated for each landmark time, capturing the patient profile up to that specific time. These are then stacked together into a “super dataset” while preserving the longitudinal structure of the data. Competing events—cancer-specific death, death from other causes, and relapse—are all considered.
✓ **RSF Model Training**: A random survival forest model is trained using the combined landmark datasets. The RSF, an ensemble of decision trees, learns from the data without assuming proportional hazards or linear relationships. The model captures complex, non-linear associations and interactions among predictors. Before model fitting, hyperparameter tuning was performed to determine the optimal mtry (number of randomly drawn candidate variables) and node size tuning parameters for a random forest, using out-of-sample error as the evaluation metric. Hyperparameter tuning for RSF was conducted using a grid search strategy. The number of candidate variables considered at each split (mtry) and the minimum node size were tuned by evaluating combinations across predefined ranges: mtry (5 to 15) and node size (5 to 50). The number of trees was fixed at 100 to ensure computational consistency. Model performance for each parameter configuration was assessed using out-of-bag (OOB) error estimates, which serve as internal out-of-sample error measures in ensemble tree models. For each tree, approximately one-third of the samples not included in the bootstrap sample were used to compute the prediction error. The optimal parameter set was defined as the one that minimized the average out-of-bag (OOB) error, measured as the OOB concordance error, across all trees.
  Given the longitudinal nature of the landmarking approach, where the same patient may contribute data at multiple time points, cluster-based resampling was employed during bootstrapping. This ensured that repeated measures for a single patient were sampled as a unit, preserving intra-subject correlation and avoiding data leakage between training and validation sets.
✓ **Model Outputs—Cumulative Incidence Functions (CIFs)**: For each patient, the RSF produces predicted cumulative incidence functions for each competing event. These CIFs quantify the probability of experiencing relapse or death at future time points, conditional on survival until the landmark. The RSF framework handled competing risks directly by estimating CIF for each event type (cancer-specific death, other-cause death, and relapse), following the methodology proposed by Ishwaran et al. [22]. This non-parametric ensemble method does not rely on Fine–Gray regression but computes CIFs by aggregating over multiple decision trees. Right-censoring was addressed using inverse probability of censoring weighting (IPCW) during tree construction, ensuring unbiased estimation of survival probabilities. Ties in survival times were resolved using standard RSF splitting rules based on ranked survival times.

The feature importance analysis has also been calculated using the minimal depth metric. Minimal depth quantifies the average distance from the root node at which a variable first splits the data across all trees in the forest. Variables that split closer to the root (i.e., with lower minimal depth) are considered more important, as they contribute earlier and more frequently to the prediction process. This method provides a relative ranking of predictors without relying on assumptions of linearity or proportional hazards. While minimal depth captures the algorithmic influence of each variable, it does not reflect effect size or direction and is therefore best interpreted in conjunction with clinical relevance.

Given the presence of multiple competing risks, traditional Kaplan–Meier survival curves were insufficient for accurately estimating event probabilities. Instead, a competing risks framework for RSF was implemented using cumulative incidence functions (CIFs), stratified at each landmark time point. This approach accounted for the fact that a patient who dies from other causes is no longer at risk for cancer-related mortality or relapse.

#### 2.2.6. Validation and Model Performance Assessment

Models A and B were validated through fivefold cross-validation. The performance of each model was assessed using Harrell’s concordance index for the Cox models and the RSF models. One hundred bootstrap-based confidence intervals were computed to quantify uncertainty and evaluate the stability of predictions.

#### 2.2.7. Patient Profiling

To visualize the dynamic behavior of cumulative risks predicted by the RSF model (as shown in Section 3.4, two illustrative patient profiles—referred to as “better” and “worse” risk groups—were constructed based on combinations of clinical variables identified as most influential in the model (i.e., those with the lowest minimal depth). These profiles were not derived through data-driven stratification but were manually defined to reflect clinically relevant contrasts in relapse risk.

The better profile represented a low-risk patient characterized by median age (65 years), male sex, medianBMI, minimal comorbidity burden (Charlson score: 0), ASA I–II status, absence of perioperative treatment, non-smoker status, elective laparoscopic approach, high tie ligation of the IMA, no anastomotic dehiscence, no transfusion, no conversion to open surgery, no combined resections, median operative time, mid-to-high tumor localization, early pathologic staging (pT0, pN0, pM0), TNM stage < II, and a median distance from the anal verge.

Conversely, the worse profile simulated a high-risk patient with the same age and sex for comparability. Still, it included high-risk features: maximum BMI, higher comorbidity burden (Charlson score: median), ASA III–IV, urgent surgical setting, open approach, low tie ligation, presence of anastomotic dehiscence, perioperative treatment, smoking, transfusion and conversion during surgery, combined resections, prolonged operative time, low tumor location, advanced pathologic staging (pT4, pN2, pM1), TNM stage > III, and minimal distance from the anal verge.

Cumulative incidence functions (CIFs) for relapse, cancer-related death, and other-cause death were estimated using the trained RSF model at each landmark time point (1, 3, and 5 years). Predictions were generated for the illustrative better and worse patient profiles by applying the model to each profile and computing event-specific CIFs, accounting for right-censoring and competing risks via ensemble averaging and inverse probability of censoring weighting (IPCW).

## 3. Results

### 3.1. Descriptive Statistics

A total of 2405 patients who underwent curative RC resection were included in the analysis. The population’s median age was 66.6 years (IQR: 58.2–74.0), with 61.7% being male. The median BMI was 25.3 kg/m^2^ (IQR: 22.7–27.6), and most patients had a Charlson comorbidity score of 2. Regarding operative risk, 74.4% of patients were classified as ASA I–II, while 25.6% were ASA III–IV. Most procedures were performed laparoscopically (56.5%), with open (35.7%) and robotic (7.8%) approaches used less frequently. Combined MVR were required in 19.7% of cases, and 11.8% experienced anastomotic dehiscence.

Tumor location was predominantly mid- (46.9%) and high-rectal (29.1%), with advanced pathological features present in a substantial proportion of the cohort, including 35.9% with TNM stage >III, 34.6% undergoing combined resections, and 7.8% with peritoneal metastases.

The median follow-up period was 6 years (IQR: 3–8 years). During this time, 187 cancer-related deaths, 474 deaths from other causes, and 202 relapses were observed (Table 1).

Cancer-related mortality was higher in older patients and those with greater comorbidity burdens, such as elevated Charlson and ASA scores. Open surgical approaches and combined MVR were also linked to an increased risk of death from cancer. As expected, advanced disease features (higher TNM stage, positive lymph nodes, and peritoneal metastases) were strongly associated with cancer-specific mortality (Table 1).

Older age and a higher Charlson score were significantly associated with increased mortality from other causes. Additional risk factors included higher ASA scores, the occurrence of anastomotic dehiscence, perioperative blood transfusions, and combined MVR. Advanced pathological features, including lymph node involvement and peritoneal metastasis, also contributed to higher mortality from other causes (Table 1).

Relapse was more frequent in patients with higher BMI, those who received perioperative treatments, and those who experienced anastomotic dehiscence. Combined MVR was also associated with a higher relapse rate. As with cancer mortality, advanced pathological staging (positive peritoneal metastases, lymph node involvement, and higher TNM stage) were major predictors of recurrence (Table 1).

### 3.2. Model A

Landmarking Cox’s proportional hazards model was applied to evaluate the influence of clinical variables on relapse risk at 1, 3, and 5 years post-surgery, capturing the dynamic nature of prognostic factors over time. The Harrell C index is 0.78 [95% bootstrap CI = 0.75–0.79] for the classical Model A.

The analysis identified combined MVR as consistently associated with an increased risk of relapse across all time points. Similarly, anastomotic dehiscence showed a relevant association with higher relapse risk, particularly in the early postoperative period, although its effect appeared to decrease over time (Table 2). A high tie ligation of the IMA was found to be protective against relapse, with a progressively stronger effect observed at later follow-up times. Distant metastases (pM1) were associated with a higher risk of relapse, significantly closer to the time of surgery, with attenuation of the effect over time.

Regarding pathological staging, lower pT stages (pT0–pT2) were associated with a reduced risk of relapse compared to pT3, with the protective effect being most pronounced in the years after surgery. The distance from the anal verge showed a time-dependent impact with a possible increase in relapse risk over time.

The open surgical approach was associated with a higher risk of relapse compared to laparoscopy, and this effect remained stable throughout the follow-up.

Other factors, such as age, BMI, Charlson score, gender, ASA classification, perioperative treatments, smoking status, conversion to open surgery, and localization of the tumor, did not show notable associations with relapse risk over time in this multivariable setting.

Overall, the landmarking analysis confirmed that relapse risk is dynamic and evolves with time since surgery, with certain risk factors having more pronounced effects at specific follow-up intervals.

### 3.3. Model B

**Figure 2:**
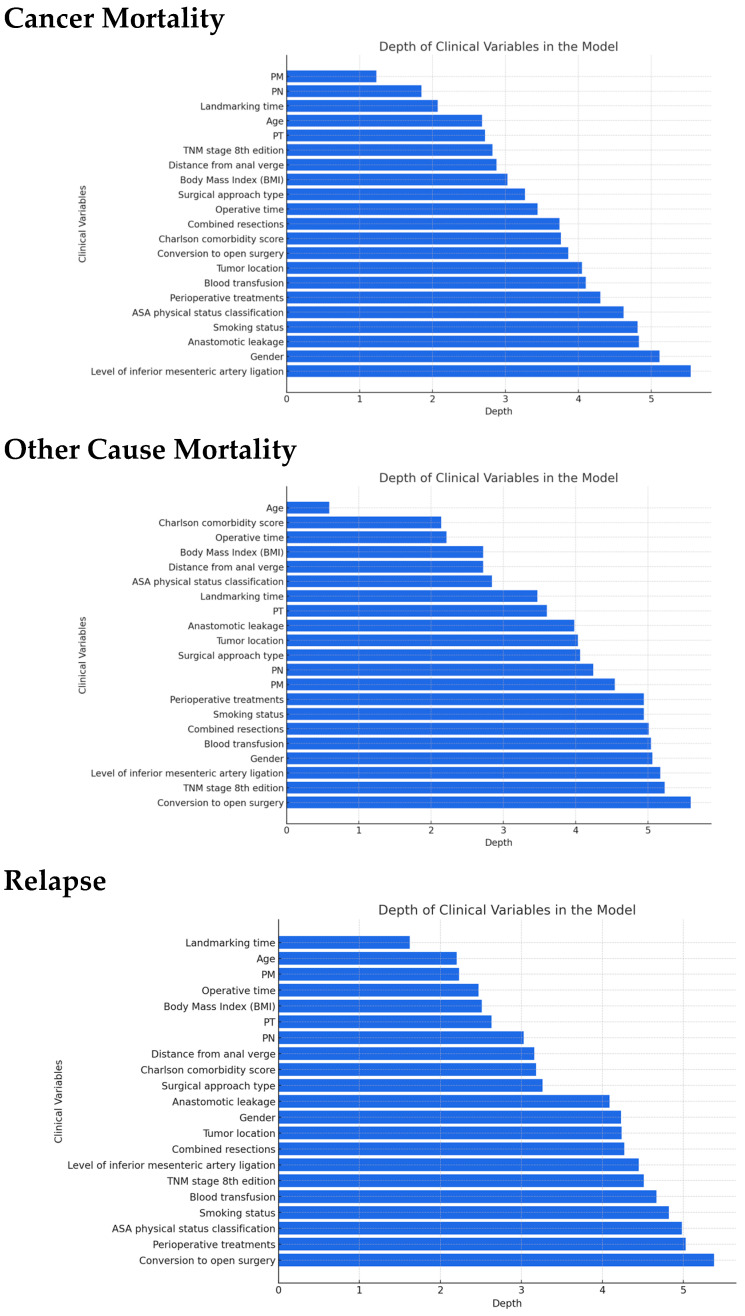
Landmark random forest. Minimal depth of clinical variables in decision tree analysis. This figure illustrates the minimal depth at which various clinical variables are used in a decision tree model, which reflects their relative importance in predicting the outcome. Each bar represents the minimal depth where a specific variable first splits the data, with a lower value indicating a higher importance in the model’s initial decision-making process. Variables are ranked from top to bottom, starting with those impacting the model as soon as possible (i.e., at the shallowest depth).

Model B’s Harrell C-index performance is notably higher than Model A’s (C index = 0.95; 95% CI = 0.82–0.96). The variable importance of clinical predictors across the three competing events (cancer-related mortality, other-cause mortality, and relapse) was evaluated using minimal depth from the random forest models. Variables with lower minimal depth appear closer to the root of the decision trees and are considered more influential for the outcome (Figure 2).

The most important variables for cancer-specific mortality were distant metastases (pM), pathological lymph node involvement (pN), landmarking time, and age. These variables had the lowest minimal depths, highlighting their significant contribution to the model’s ability to predict cancer-related death. Pathological features such as TNM stage, distance from the anal verge, and BMI also showed relevance. Conversely, variables like gender, anastomotic leakage, and the level of IMA ligation were less influential in this model (Figure 2).

In the model for mortality from other causes, age was the dominant predictor, followed by Charlson comorbidity score and operative time, reflecting the relevance of general health status and surgical complexity for non-cancer-related death. Variables related to tumor characteristics (e.g., pT, pN, pM) and perioperative factors (e.g., anastomotic leakage, blood transfusion) contributed less, indicating that patient frailty outweighed tumor burden in predicting other-cause mortality (Figure 2).

Landmarking time, age, and distant metastases (pM) were the most influential variables for relapse prediction. Tumor burden indicators such as pathological tumor stage (pT) and pathological node stage (pN) also played important roles. Notably, combined MVR, surgical approach, and perioperative complications such as anastomotic leakage had a more modest contribution in this setting (Figure 2).

### 3.4. Patient Profiling

**Figure 3:**
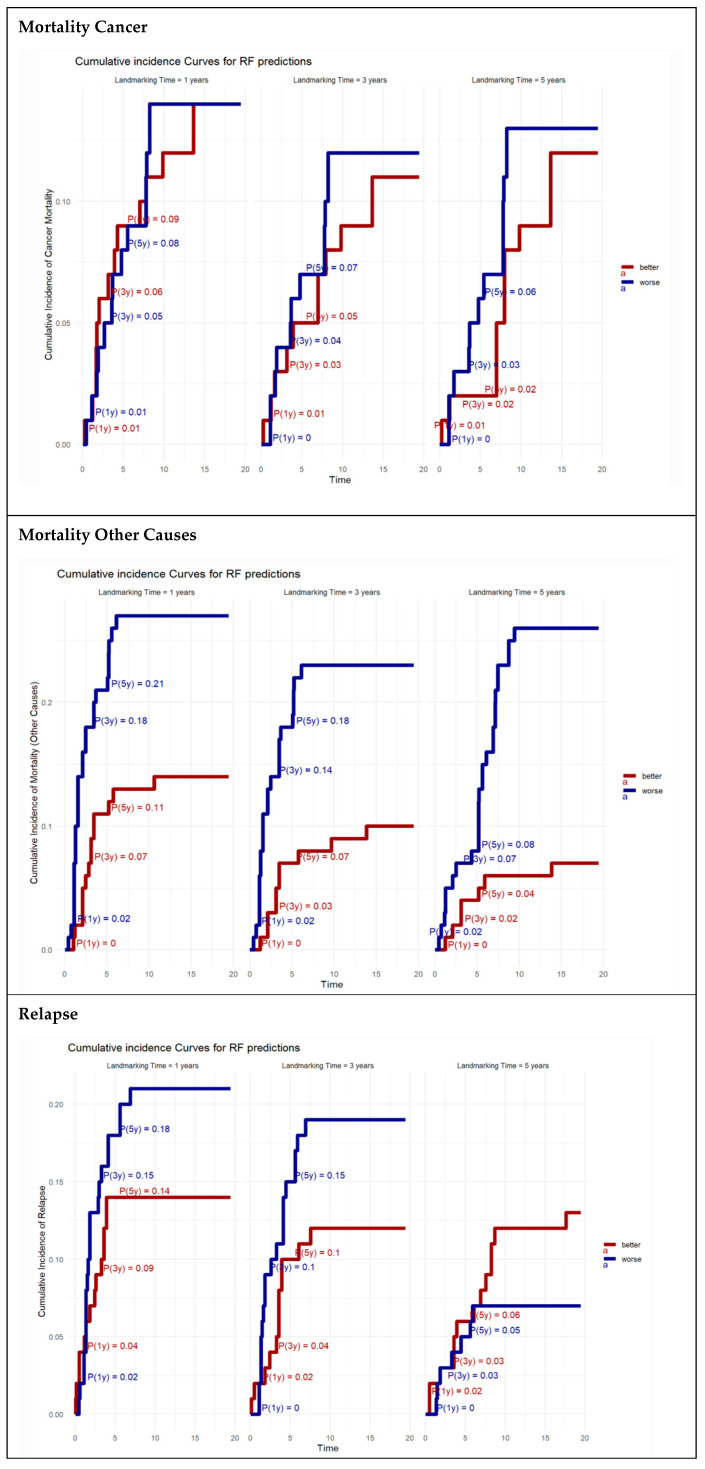
Random forest landmark cumulative incidence curves for cancer mortality predictions, mortality for other causes, and relapse. This figure presents the cumulative incidence of cancer mortality predicted by random forest models for landmarking times of 1, 3, and 5 years. Each panel displays curves for two groups classified as “better” (blue) and “worse” (red) based on their risk profiles. The curves illustrate the probability of cancer mortality over time, with annotations showing the probability at specific years, highlighting the differences in outcomes between the groups at these times. The numeric values on the curves represent the cumulative probabilities of mortality, providing insights into the effectiveness of the predictive model over extended periods.

Figure 3 shows the cumulative incidence functions (CIFs) predicted by the RSF model for cancer-specific mortality, other-cause mortality, and relapse at landmark times of 1, 3, and 5 years. At all time points, predicted cumulative incidence was higher in the worse profile compared to the better profile for each of the three outcomes.

For cancer-specific mortality, the cumulative incidence increased progressively with time and was consistently higher in the worse profile. The difference in predicted risk between the profiles was evident at each landmark and became more pronounced at later time points.

For other-cause mortality, predicted risk also increased over time in both profiles, with a greater rise observed in the worse profile. The curves showed more gradual slopes compared to cancer-specific mortality, with less pronounced differences between profiles.

For a relapse, the predicted cumulative incidence rose rapidly at earlier time points and plateaued more gradually at later landmarks. The worse profile exhibited higher predicted relapse probabilities than the better profile across all landmarks.

### 3.5. Models Comparison

The two modeling approaches demonstrated apparent differences in performance and capabilities. Model A, a traditional cause-specific Cox proportional hazards model with landmarking, achieved a Harrell C-index of 0.78 (95% CI: 0.75–0.79), reflecting moderate discriminative ability. In contrast, Model B, based on a random survival forest (RSF) algorithm within the same landmarking framework, achieved superior predictive performance, with a Harrell C-index of 0.95 (95% CI: 0.82–0.96). While Model A offers high interpretability through hazard ratios, it assumes proportional hazards and linear relationships, which may limit its flexibility in capturing complex interactions. Model B, being non-parametric, efficiently handled non-linear effects and variable interactions without restrictive assumptions. In the Cox model, no formal interaction terms were introduced, as the focus was on estimating time-dependent effects within the landmarking framework. In contrast, the RSF model implicitly captures non-linear effects and complex interactions through recursive partitioning, without requiring pre-specification of interaction terms. While no formal statistical testing of interactions was conducted.

Additionally, Model B explicitly modeled competing risks using cumulative incidence functions, enhancing its applicability in the presence of cancer-related and other-cause mortality. Model B outperformed Model A regarding discrimination, adaptability, and ability to incorporate dynamic, time-varying risk profiles (Table 3).

## 4. Discussion

The relative survival of patients with RC has improved considerably over the years, especially in more advanced tumor stages. The 5-year survival rate increased for all RC stages combined from 51% to 65% [25]. In the present study, the 5-year overall survival (OS) is 63%, which aligns with literature data. The early detection of relapse at a treatable stage is the main objective of follow-up after curative surgery for RC. Thus, early diagnosis of recurrent RC can increase patient eligibility for many effective treatments, reduce morbidity, and improve overall survival.

Risk assessment of relapse is crucial for early decision-making; several disease-, operative-, and postoperative-related issues should be considered, including the type of approach, combined MVR, level of IMA ligation, locally advanced tumor, lymph-node metastases, distant metastases, and anastomotic leakage.

The open approach resulted in a risk factor for cancer relapse as compared with laparoscopic surgery. Although a very recent RCT from China [26], investigating the non-inferiority of laparoscopic surgery versus the open approach, reported an increased 3-year locoregional recurrence in the open arm compared with the laparoscopic arm (3.7% vs. 2.3%; *p* = 0.22), our results did not align with most of the literature evidence. Specifically, several systematic reviews and meta-analyses [27,28] have shown similar short- and long-term oncological outcomes between the laparoscopic and open approaches. We can justify our findings by suggesting that open surgery was likely administered to more challenging cases, such as those involving locally advanced cancers, tumors invading nearby organs, metastatic disease, and intraoperative adverse events, all of which are widely recognized risk factors for cancer relapse [27].

We were unable to find a study investigating the oncological outcomes of locally advanced RC underwent MVR. However, MVR is usually performed for rectal tumors invading nearby organs (T4b) that are widely recognized as an independent factor for local recurrence [28], corroborating our results.

In the present study, the low tie ligation of IMA is a risk factor for cancer relapse as compared with high ligation. The ligation of the IMA is one of the most relevant procedures during anterior resection for RC to ensure adequate lymphadenectomy and colon mobilization. Compelling evidence suggests that lymph node dissection is mandatory to reduce the risk of local recurrence [29,30,31]. Some authors argue that low ligation of the IMA does not yield sufficient lymph nodes for oncological safety [32]. In contrast, others contend that routine high tie dissection is unnecessary due to the relatively uncommon occurrence of lymph node metastases at the root of the IMA [33,34]. To date, no consensus has been reached regarding the optimal level of IMA ligation in terms of postoperative complications, quality of life, and oncological outcomes. Our finding adds to the ongoing debate surrounding inferior mesenteric artery ligation.

To our knowledge, a more advanced cancer stage is strictly related to a worse prognosis. Thirty percent of patients with RC stages I–III develop tumor relapse, while up to 65% of patients with stage IV RC experience recurrent disease after curative treatment [27,35].

Finally, a recent meta-analysis, which included 34,487 patients who underwent curative anterior resection for RC, concluded that anastomotic leakage is associated with increased local recurrence and reduced long-term survival [36]. In alignment with the literature data, the present study identified anastomotic leakage after surgery as a leading risk factor for cancer relapse.

To our knowledge, this is the first study to propose an ML algorithm for predicting the risk of relapse, which changes over time after surgery. This is consistent with the new concept of conditional survival. Zheng et al. [12] conducted a retrospective study on 785 patients treated for RC to create CS nomograms that predicted the conditional probability of survival after rectal resection. The author demonstrated that the likelihood of achieving 5-year recurrence-free survival increased from 75.6% immediately after surgery to 93.9% in patients who had already survived for 3 years without recurrence. CS provides a more tailored prognosis over time and helps to calibrate postoperative surveillance strategies. Besides, the CS analysis has already been adopted as a predictor for the risk of relapse in other types of cancer, such as breast [37], hemopoietic [38], liver [39], and esophageal [40].

To date, several professional societies have proposed imaging-based surveillance guidelines for RC. These guidelines are consistent in recommendations for surveillance every 3–6 months for the first 2–3 years after surgery, then every 6 months for a total of 5 years with clinical examination, imaging, and colonoscopy (after 1 year) [6,7,8]. Despite these differences in the frequency and type of surveillance, there is consensus that overly intensive follow-up does not necessarily yield additional benefits. Additional radiation exposure from surveillance tests could pose risks of developing other types of tumors [41]. Furthermore, it’s essential to consider the phenomenon of “scanxiety,” a term widely adopted to describe the psychosocial effects of routine surveillance imaging on cancer survivors, which can provoke anxiety and distress in many of them [42].

The findings of this study underscore the value of landmarking as a methodological framework for survival prediction. Landmarking enhances prognostic accuracy and facilitates personalized clinical decision-making by enabling the reassessment of risk factors at multiple time points. Patients do not have a fixed survival probability at the time of surgery; their risk changes as they progress through different stages of follow-up.

Landmarking is particularly useful in oncology, where patients in the early stages have significantly different survival trajectories from those who remain event-free for several years. By updating risk estimates dynamically, clinicians can provide more precise prognostic information and tailor follow-up strategies accordingly. This approach has broader applicability beyond colorectal cancer, extending to other chronic diseases where time-dependent risk assessment is critical.

The integration of landmarking with machine learning-based RSF models provides a flexible and robust framework for survival prediction. By dynamically updating relapses over time and incorporating complex, non-linear interactions among clinical variables, this approach supports more personalized follow-up strategies, enabling the identification of high-risk patients who may benefit from intensified monitoring, while sparing low-risk individuals from unnecessary interventions. Nonetheless, the traditional Cox model remains clinically relevant in scenarios where transparency and interpretability are paramount. For example, when informing guideline development, when communicating prognosis with patients, or in settings with limited computational resources or smaller datasets, the Cox model provides precise hazard ratio estimates that are familiar to clinicians and easier to apply in routine decision-making. In our analysis, the superior performance of the model compared to the widely used ANN model highlights RSF’s effectiveness in capturing certain variable relationships within clinical data—relationships that the ANN does not handle as efficiently in this specific context. ANNs are powerful models; they often require larger datasets with complex big data [43,44].

The clinical relevance of our approach lies in its potential to tailor personalized postoperative surveillance. For instance, patients identified as high-risk at a given time point can be prioritized for more intensive imaging and monitoring. In contrast, low-risk patients may be safely followed with less frequent testing, thereby improving resource utilization and patient experience. Beyond improving prognostic accuracy, this model could support more cost-effective follow-up strategies. Current surveillance protocols often apply uniform schedules regardless of individual relapse risk, which can result in unnecessary imaging, laboratory tests, and outpatient visits for low-risk patients while potentially under-monitoring those at higher risk.

By identifying patients with persistently low relapse probabilities, such as those with favorable profiles, follow-up intensity can be safely reduced, thereby lowering healthcare costs and minimizing patient burden from frequent testing and hospital visits. Conversely, intensified surveillance might facilitate earlier detection of recurrence and more timely interventions for high-risk patients, potentially improving outcomes without proportionally increasing costs.

In the context of existing literature, follow-up schemes have been reported to vary widely in cost, ranging from $910 to $26,717 over 5 years, depending on the intensity [9]. A tailored strategy based on our model could optimize resource allocation by concentrating on healthcare efforts where they are most needed. Future prospective studies could simulate the economic impact of risk-adapted surveillance protocols by comparing the cumulative costs and outcomes of standard vs. model-driven follow-up approaches.

This study has many limitations due to its retrospective design and relatively long accrual period. Several issues had important missing data, with a consequently limited number of factors analyzed. Furthermore, many changes concerning perioperative and operative treatment characterized this long accrual interval.

While the RSF model offers the advantage of capturing complex interactions and nonlinear effects, its internal measures of variable importance, such as minimal depth, should be interpreted with caution. These rankings are unitless and algorithm-driven, reflecting how early and frequently a variable is used to split nodes across decision trees. Still, they do not provide quantitative estimates of effect size or direct measures of clinical risk. Therefore, the most influential variables for predicting relapse and mortality must be understood in the context of existing clinical evidence and pathophysiology. For example, the prominence of pM and pN status in the model aligns with their well-established prognostic roles in rectal cancer. Ultimately, variable importance metrics are helpful in exploring model behavior, but clinical applicability relies on integrating these results with domain knowledge and individual patient profiles.

Moreover, internal validation through bootstrapping confirms the robustness of the model; external validation with independent datasets should also be important to evaluate its generalizability across diverse ICU settings and patient populations. The modeling framework presented in this study has the potential to support risk-adapted post-operative surveillance in rectal cancer. By integrating time-dependent clinical information and competing risks, the RSF model enables dynamic and individualized prediction of relapse and mortality. This can inform more precise follow-up strategies, such as intensifying imaging schedules for high-risk patients or safely de-escalating surveillance in low-risk cases, ultimately improving clinical decision-making and resource allocation.

To facilitate clinical integration, future work will focus on external validation of the model in independent cohorts from other institutions or population-based registries. Additionally, efforts will be made to translate the model into a user-friendly platform, allowing clinicians to input patient characteristics and receive real-time risk predictions.

## 5. Conclusions

In this era of precision medicine, more tailored surveillance for patients undergoing curative surgery for rectal cancer is needed to obtain early diagnosis of relapses, reduce the cost of ineffective tests, minimize radiation exposure risks, and alleviate negative psychosocial effects. In this study, we analyzed and compared the importance of risk factors for rectal cancer relapse using a machine-learning algorithm. The clinical relevance of our approach lies in its potential to tailor personalized postoperative surveillance.

## Figures and Tables

**Table 1:** Survival outcomes and clinical characteristics of patients. The table presents cause-specific survival outcomes and clinical characteristics for a cohort of patients, differentiated by outcomes such as death due to cancer, death due to other causes, and relapse. The table includes counts and percentages for each categorical variable, median, and IQR for quantitative variables with cause-specific hazard ratios and *p*-values.

		Death for Cancer				Death for Other Causes				Relapse			
Variables	Overall	No Events	Event	HR	P	No Events	Event	HR	P	No Events	Event	HR	P
	N = 2405	N = 2218	N = 187			N = 1931	N = 474			N = 2203	N = 202		
**Age (years)**	66.6 [58.2;74.0]	66.4 [58.0;73.9]	69.3 [61.3;75.8]	1.03 [1.01;1.04]	<0.001	64.4 [56.3;70.8]	74.6 [68.5;79.6]	1.09 [1.08;1.10]	<0.001	66.7 [58.2;74.1]	66.4 [56.4;73.9]	1.00 [0.99;1.01]	0.737
**Gender:**					0.171				0.001				0.096
Female	921 (38.3%)	856 (38.6%)	65 (34.8%)	Ref.		768 (39.8%)	153 (32.3%)	Ref.		852 (38.7%)	69 (34.2%)	Ref.	
Male	1484 (61.7%)	1362 (61.4%)	122 (65.2%)	1.23 [0.91;1.67]		1163 (60.2%)	321 (67.7%)	1.40 [1.16;1.70]		1351 (61.3%)	133 (65.8%)	1.28 [0.96;1.71]	
**BMI**	25.3 [22.7;27.6]	25.4 [22.8;27.7]	24.9 [22.0;27.2]	0.96 [0.92;1.00]	0.081	25.3 [22.7;27.6]	25.4 [22.9;27.5]	1.01 [0.97;1.04]	0.726	25.2 [22.6;27.5]	26.1 [24.0;29.0]	1.06 [1.01;1.11]	0.012
**Charlson Comorbidity Score**	2.00 [2.00;3.00]	2.00 [2.00;3.00]	2.00 [2.00;3.00]	1.20 [1.05;1.37]	0.008	2.00 [2.00;3.00]	3.00 [2.00;3.00]	1.42 [1.32;1.54]	<0.001	2.00 [2.00;3.00]	2.00 [2.00;3.00]	1.16 [0.99;1.35]	0.058
**ASA:**					0.011				<0.001				0.42
I–II	1515 (74.4%)	1410 (75.0%)	105 (67.3%)	Ref.		1283 (78.1%)	232 (58.9%)	Ref.		1383 (74.5%)	132 (73.7%)	Ref.	
III–IV	521 (25.6%)	470 (25.0%)	51 (32.7%)	1.54 [1.10;2.15]		359 (21.9%)	162 (41.1%)	2.19 [1.79;2.68]		474 (25.5%)	47 (26.3%)	1.15 [0.82;1.60]	
**Perioperative treatments:**					0.158				0.001				0.01
No	287 (19.7%)	258 (19.4%)	29 (22.0%)	Ref.		227 (18.9%)	60 (23.3%)	Ref.		276 (20.8%)	11 (8.33%)	Ref.	
Yes	1172 (80.3%)	1069 (80.6%)	103 (78.0%)	0.74 [0.49;1.12]		974 (81.1%)	198 (76.7%)	0.61 [0.46;0.82]		1051 (79.2%)	121 (91.7%)	2.20 [1.19;4.09]	
**Smoke:**					0.886				0.425				0.903
No	1156 (75.8%)	1053 (75.9%)	103 (74.6%)	Ref.		975 (76.1%)	181 (74.2%)	Ref.		1060 (75.7%)	96 (76.2%)	Ref.	
Yes	370 (24.2%)	335 (24.1%)	35 (25.4%)	1.03 [0.70;1.51]		307 (23.9%)	63 (25.8%)	1.12 [0.84;1.50]		340 (24.3%)	30 (23.8%)	0.97 [0.65;1.47]	
**Surgical approach:**					<0.001				0.026				0.682
Laparoscopic	1312 (56.5%)	1242 (58.1%)	70 (38.5%)	Ref.		1078 (57.5%)	234 (52.6%)	Ref.		1208 (56.7%)	104 (54.7%)	Ref.	
Open	828 (35.7%)	734 (34.3%)	94 (51.6%)	1.93 [1.41;2.63]		633 (33.7%)	195 (43.8%)	1.02 [0.84;1.24]		754 (35.4%)	74 (38.9%)	0.95 [0.70;1.29]	
Robotic	181 (7.80%)	163 (7.62%)	18 (9.89%)	1.65 [0.98;2.77]		165 (8.80%)	16 (3.60%)	0.51 [0.31;0.85]		169 (7.93%)	12 (6.32%)	0.77 [0.42;1.40]	
**Inferior mesenteric artery ligation level:**					0.085				0.647				0.373
High tie	1752 (85.3%)	1583 (84.8%)	169 (90.4%)	Ref.		1450 (85.2%)	302 (85.8%)	Ref.		1617 (85.1%)	135 (88.2%)	Ref.	
Low tie	301 (14.7%)	283 (15.2%)	18 (9.63%)	0.65 [0.40;1.06]		251 (14.8%)	50 (14.2%)	0.93 [0.69;1.26]		283 (14.9%)	18 (11.8%)	0.80 [0.49;1.31]	
**Anastomotic dehiscence:**					0.906				<0.001				0.001
No	2121 (88.2%)	1954 (88.1%)	167 (89.3%)	Ref.		1723 (89.2%)	398 (84.0%)	Ref.		1955 (88.7%)	166 (82.2%)	Ref.	
Yes	284 (11.8%)	264 (11.9%)	20 (10.7%)	0.97 [0.61;1.55]		208 (10.8%)	76 (16.0%)	1.67 [1.31;2.13]		248 (11.3%)	36 (17.8%)	1.79 [1.25;2.57]	
**Combined multivisceral resections:**					<0.001				0.037				0.006
No	1676 (80.3%)	1555 (81.7%)	121 (65.4%)	Ref.		1390 (80.8%)	286 (77.9%)	Ref.		1559 (80.8%)	117 (73.6%)	Ref.	
Yes	412 (19.7%)	348 (18.3%)	64 (34.6%)	2.40 [1.77;3.25]		331 (19.2%)	81 (22.1%)	1.30 [1.02;1.67]		370 (19.2%)	42 (26.4%)	1.64 [1.15;2.34]	
**Operative time (min):**	240 [180;300]	240 [180;300]	240 [195;300]	1.00 [1.00;1.00]	0.809	240 [180;300]	240 [180;300]	1.00 [1.00;1.00]	0.033	240 [180;300]	250 [192;312]	1.00 [1.00;1.00]	0.029
**Trasfusion:**					0.066				<0.001				0.009
No	1949 (91.6%)	1805 (91.8%)	144 (89.4%)	Ref.		1575 (92.5%)	374 (88.0%)	Ref.		1782 (91.9%)	167 (88.8%)	Ref.	
Yes	179 (8.41%)	162 (8.24%)	17 (10.6%)	1.60 [0.97;2.64]		128 (7.52%)	51 (12.0%)	2.24 [1.67;3.01]		158 (8.14%)	21 (11.2%)	1.81 [1.15;2.86]	
**Conversion:**					0.862				0.167				0.617
No	1835 (94.2%)	1707 (94.3%)	128 (93.4%)	Ref.		1452 (94.0%)	383 (95.0%)	Ref.		1684 (94.3%)	151 (92.6%)	Ref.	
Yes	113 (5.80%)	104 (5.74%)	9 (6.57%)	1.06 [0.54;2.09]		93 (6.02%)	20 (4.96%)	0.73 [0.47;1.14]		101 (5.66%)	12 (7.36%)	1.16 [0.64;2.09]	
**AV distance:**	8.00 [6.00;11.0]	8.80 [6.00;11.0]	8.00 [6.00;11.0]	1.00 [0.96;1.04]	0.987	8.00 [6.00;11.0]	10.0 [6.00;12.0]	1.05 [1.03;1.08]	<0.001	8.00 [6.00;11.0]	9.00 [6.00;11.0]	1.03 [0.99;1.07]	0.161
**Localization:**					0.558				0.027				0.883
High	682 (29.1%)	632 (29.2%)	50 (27.9%)	Ref.		532 (28.0%)	150 (33.6%)	Ref.		627 (29.2%)	55 (27.8%)	Ref.	
Middle	1100 (46.9%)	1011 (46.7%)	89 (49.7%)	1.09 [0.77;1.55]		903 (47.5%)	197 (44.2%)	0.80 [0.65;0.99]		1005 (46.8%)	95 (48.0%)	1.05 [0.76;1.47]	
Low	564 (24.0%)	524 (24.2%)	40 (22.3%)	0.89 [0.59;1.35]		465 (24.5%)	99 (22.2%)	0.72 [0.56;0.93]		516 (24.0%)	48 (24.2%)	0.97 [0.66;1.43]	Low
**(y)pT:**					<0.001				<0.001				<0.001
0	223 (9.55%)	215 (10.0%)	8 (4.28%)	Ref.		187 (9.95%)	36 (7.89%)	Ref.		214 (10.0%)	9 (4.48%)	Ref.	
1	316 (13.5%)	307 (14.3%)	9 (4.81%)	0.74 [0.29;1.93]		263 (14.0%)	53 (11.6%)	0.88 [0.57;1.34]		304 (14.2%)	12 (5.97%)	0.84 [0.36;2.01]	
2	589 (25.2%)	565 (26.3%)	24 (12.8%)	1.14 [0.51;2.54]		481 (25.6%)	108 (23.7%)	1.06 [0.73;1.55]		553 (25.9%)	36 (17.9%)	1.48 [0.71;3.07]	
3	1079 (46.2%)	957 (44.6%)	122 (65.2%)	3.73 [1.82;7.63]		850 (45.2%)	229 (50.2%)	1.62 [1.14;2.31]		960 (45.0%)	119 (59.2%)	3.27 [1.66;6.44]	
4	128 (5.48%)	104 (4.84%)	24 (12.8%)	7.19 [3.23;16.0]		98 (5.22%)	30 (6.58%)	2.05 [1.26;3.33]		103 (4.83%)	25 (12.4%)	6.77 [3.16;14.5]	
**(y)pM:**					<0.001				0.001				<0.001
0	2188 (92.2%)	2060 (94.3%)	128 (68.4%)	Ref.		1755 (92.0%)	433 (93.3%)	Ref.		2030 (93.5%)	158 (78.6%)	Ref.	
1	184 (7.76%)	125 (5.72%)	59 (31.6%)	10.6 [7.77;14.5]		153 (8.02%)	31 (6.68%)	1.86 [1.29;2.69]		141 (6.49%)	43 (21.4%)	6.86 [4.87;9.65]	
**(y)pN:**					<0.001				0.001				<0.001
0	1551 (65.4%)	1489 (68.1%)	62 (33.2%)	Ref.		1243 (65.2%)	308 (66.0%)	Ref.		1452 (66.8%)	99 (49.5%)	Ref.	
1	545 (23.0%)	473 (21.6%)	72 (38.5%)	3.81 [2.72;5.36]		439 (23.0%)	106 (22.7%)	1.20 [0.96;1.49]		489 (22.5%)	56 (28.0%)	1.92 [1.38;2.67]	
2	277 (11.7%)	224 (10.2%)	53 (28.3%)	7.19 [4.97;10.4]		224 (11.8%)	53 (11.3%)	1.72 [1.28;2.31]		232 (10.7%)	45 (22.5%)	4.16 [2.91;5.94]	
**Stage (y)pTNM 8th:**					<0.001				0.013				<0.001
<II	1541 (64.1%)	1485 (67.0%)	56 (29.9%)	Ref.		1233 (63.9%)	308 (65.0%)	Ref.		1447 (65.7%)	94 (46.5%)	Ref.	
>III	864 (35.9%)	733 (33.0%)	131 (70.1%)	5.16 [3.77;7.06]		698 (36.1%)	166 (35.0%)	1.27 [1.05;1.54]		756 (34.3%)	108 (53.5%)	2.64 [2.00;3.49]	

BMI: body mass index; ASA: American Society of Anesthesiologists; min: minutes; AV: anal verge; (y)pT, pathological T stage according to the 8th edition of the TNM classification after neoadjuvant treatment (Y) when administered; (y)pN, pathological N stage according to the 8th edition of the TNM classification after neoadjuvant treatment (Y) when administered; pM, pathological M stage according to the 8th edition of the TNM classification; (y)pTNM, pathological TNM stage according to the 8th edition of the TNM classification after neoadjuvant treatment (Y) when administered; HR: hazard ratio; P: *p*-value.

**Table 2:** Landmark Cox model relapse specific hazard ratios (HRs) and 95% confidence intervals (CIs) for various clinical factors at 1, 3, and 5 years. The Harrell C index is 0.78 [95% bootstrap CI = 0.75–0.79].

Variables	1 Year HR (95% CI)	3 Years HR (95% CI)	5 Years HR (95% CI)	Global Effect	Interaction with Time
Age	1.12 (0.89–1.41)	0.96 (0.76–1.22)	0.83 (0.64–1.09)	0.08	<0.001
BMI	1.15 (0.96–1.37)	1.15 (0.96–1.38)	1.15 (0.94–1.40)	0.06	0.99
Charlson Comorbidity Score	1.05 (0.91–1.23)	0.99 (0.83–1.17)	0.92 (0.74–1.14)	0.28	0.06
Operative Time	1.10 (0.88–1.37)	1.05 (0.81–1.35)	1.00 (0.74–1.35)	0.74	0.31
Transfusion	1.60 (0.97–2.64)	1.33 (0.73–2.41)	1.11 (0.51–2.43)	0.06	0.2
Conversion	0.78 (0.42–1.42)	0.92 (0.50–1.71)	1.09 (0.53–2.23)	0.42	0.16
AV Distance	1.68 (0.90–3.14)	1.33 (0.68–2.58)	1.05 (0.49–2.26)	0.07	0.04
Landmarking Time	0.40 (0.25–0.63)			0.02	
Gender—F:M	0.84 (0.60–1.18)	0.85 (0.60–1.22)	0.86 (0.57–1.31)	0.32	0.85
ASA—III-IV:I-II	0.97 (0.66–1.43)	0.91 (0.59–1.40)	0.85 (0.50–1.43)	0.93	0.42
Perioperative Treatments—No:Yes	0.77 (0.49–1.20)	0.78 (0.47–1.31)	0.80 (0.42–1.53)	0.47	0.84
Smoking—Yes:No	1.07 (0.74–1.55)	0.99 (0.66–1.49)	0.91 (0.56–1.49)	0.81	0.28
Surgical Approach—Open: Laparoscopic	0.59 (0.41–0.85)	0.58 (0.40–0.85)	0.57 (0.37–0.89)	0.03	0.48
Surgical Approach—Robotic: Laparoscopic	0.91 (0.48–1.75)	1.07 (0.52–2.19)	1.25 (0.52–3.00)	0.91	0.4
Lower Mesenteric Artery Ligation—Low:High	0.85 (0.50–1.47)	0.47 (0.24–0.91)	0.26 (0.11–0.61)	<0.001	<0.001
Anastomotic Dehiscence—Yes:No	1.53 (1.01–2.31)	1.56 (0.99–2.48)	1.60 (0.91–2.81)	0.02	0.13
Combined Multivisceral Resections—Yes:No	1.22 (0.80–1.86)	1.53 (1.00–2.35)	1.93 (1.20–3.09)	0.01	<0.001
Localization—High:Middle	0.59 (0.33–1.04)	0.68 (0.37–1.25)	0.78 (0.39–1.60)	0.07	0.34
Localization—Low:Middle	1.33 (0.70–2.52)	1.15 (0.58–2.27)	0.99 (0.44–2.20)	0.1	0.26
(y)pT—0:3	0.35 (0.16–0.77)	0.50 (0.23–1.08)	0.71 (0.31–1.66)	<0.001	0.002
(y)pT—1:3	0.41 (0.22–0.77)	0.54 (0.28–1.02)	0.71 (0.35–1.41)	0.001	0.02
(y)pT—2:3	0.55 (0.35–0.86)	0.58 (0.37–0.93)	0.62 (0.37–1.06)	0.01	0.28
(y)pT—4:3	1.41 (0.82–2.42)	1.24 (0.67–2.29)	1.09 (0.51–2.36)	0.12	0.37
(y)pM—1:0	4.42 (2.60–7.53)	3.00 (1.52–5.92)	2.03 (0.81–5.10)	<0.001	0.02
(y)pN—1:0	1.40 (0.65–3.02)	1.46 (0.68–3.13)	1.53 (0.68–3.47)	0.28	0.99
(y)pN—2:0	2.53 (1.13–5.68)	2.85 (1.24–6.55)	3.22 (1.24–8.32)	0.03	0.53
(y)pTNM Stage—>III:<II	0.77 (0.35–1.68)	0.61 (0.28–1.31)	0.48 (0.21–1.09)	0.54	0.15

CI: 95% confidence intervals; BMI: body mass index; ASA: American Society of Anesthesiologists; min: minutes; AV: anal verge; F: female; M: male; (y)pT, pathological T stage according to the 8th edition of the TNM classification after neoadjuvant treatment (Y) when administered; (y)pN, pathological N stage according to the 8th edition of the TNM classification after neoadjuvant treatment (Y) when administered; pM, pathological M stage according to the 8th edition of the TNM classification; (y)pTNM, pathological TNM stage according to the 8th edition of the TNM classification after neoadjuvant treatment (Y) when administered; HR: hazard ratios. Model B.

**Table 3:** Summary comparison of the two predictive models (Model A: Cause-Specific Cox with Landmarking; Model B: Random Survival Forest with Landmarking) used for dynamic relapse and survival prediction in rectal cancer patients. The table outlines key methodological features, assumptions, performance metrics, and interpretability considerations to highlight the strengths and limitations of each approach.

Feature	Model A (Cause-Specific Cox + Landmarking)	Model B (Random Survival Forest + Landmarking)
Model Type	Traditional Cox proportional hazards model	Machine learning random survival forest
Time-dependency Handling	Handled via landmarking at fixed time points	Handled via landmarking at fixed time points
Assumptions	Proportional hazards, linearity	Non-parametric, no proportional hazards assumption
Outcome Type	Relapse, with cancer and other-cause death as competing risks	Relapse, with cancer and other-cause death as competing risks
Validation Method	fivefold cross-validation, bootstrap CI	fivefold cross-validation, bootstrap CI
Performance Metric	Harrell Concordance Index	Harrell Concordance Index
Harrell C-Index	0.78 [95% CI: 0.75–0.79]	0.95 [95% CI: 0.82–0.96]
Interpretability	High (hazard ratios)	Moderate (feature importance via minimal depth)
Ability to Capture Non-linear Effects	Limited	High
Competing Risks Framework	Handled indirectly through a cause-specific approach	Explicitly modeled with cumulative incidence functions

A sensitivity analysis using an artificial neural network (ANN) model was performed, demonstrating lower performance compared to the RF algorithm, with a C-index of 0.7 (0.69–0.72); other details are reported in the Supplementary Materials.

## Data Availability

The data can be shared up on request.

## Supplementary Materials

The following supporting information can be downloaded at https://www.mdpi.com/article/10.3390/cancers17081294/s1, Figure S1: Representation of the machine learning model used in this study: a Random Survival Forest (RSF) integrated with a landmarking approach.

## Abbreviations

The following abbreviations are used in this manuscript:

RC	rectal cancer
PME	partial mesorectal excision
TME	total mesorectal excision
CRT	chemoradio therapy
CT	chemotherapy
RCT	randomized controlled trial
CS	conditional survival
AI	artificial intelligence
ML	machine learning
BMI	body mass index
MVR	multivisceral resection
CRC	colorectal cancer
HR	hazard ratio
RF	random forest
