# Supplementary material for: Dynamic Prediction of Rectal Cancer Relapse and Mortality Using a Landmarking-Based Machine Learning Model: A Multicenter Retrospective Study from the Italian Society of Surgical Oncology—Colorectal Cancer Network Collaborative Group
Source: Cancers (Basel). 2025 Apr 11;17(8):1294. doi: 10.3390/cancers17081294 (PMC12025494; doi:10.3390/cancers17081294)
Supplement: Supplementary file 1 [file cancers-17-01294-s001.zip › cancers-3536766-supplementary.pdf]

Supplement

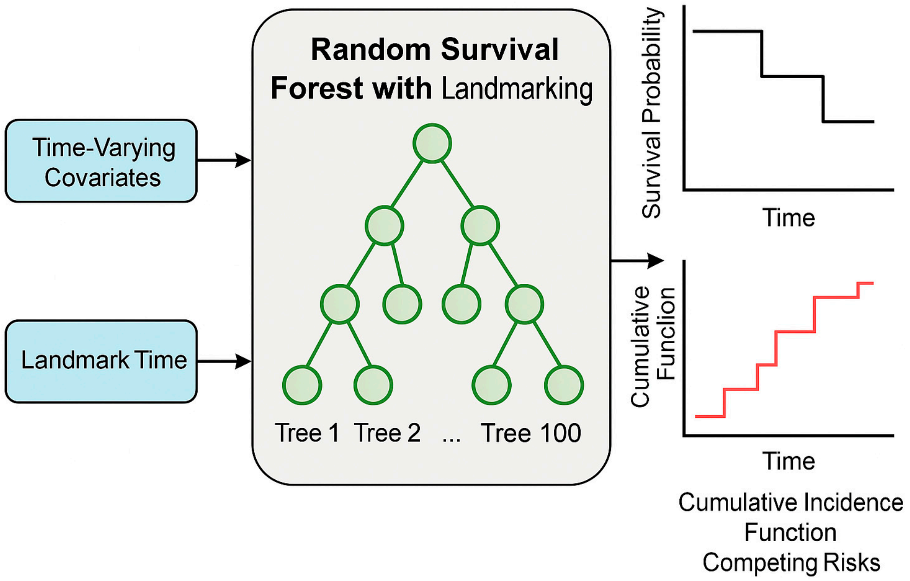

Figure S1 Representation of the machine learning model used in this study: a Random Survival Forest (RSF) integrated with a landmarking approach. Time-varying clinical covariates and predefined landmark times are used to construct landmark-specific datasets. These are then processed by the RSF model to estimate survival probabilities and cumulative incidence functions while accounting for competing risks. The model captures non-linear relationships and dynamically updates risk predictions over time to support individualized clinical decision-making.

Artificial Neural Network

A sensitivity analysis was performed by considering the Artificial Neural Network (ANN).

The survival analysis was performed using a neural network-based Cox proportional hazards model, implemented with the pycox Python library. The neural network architecture was defined as a fully connected feed-forward model with three linear layers. The input consisted of standardized clinical and demographic features (numerical and one-hot encoded categorical variables).

The architecture is as follows:

- First layer: Linear (32 units), followed by ReLU activation and batch normalization;
- Second layer: Linear (32 units), ReLU, and batch normalization;
- Final layer: Linear (1 unit), producing a single log-risk output.

The model was optimized using the Adam optimizer with a batch size of 8 and up to 100 training epochs. An early stopping strategy was applied based on the validation loss to reduce the risk of overfitting. The loss function minimized was the negative partial log-likelihood from the Cox proportional hazards framework.

ANN-based models have demonstrated lower predictive performance with a C-index of 0.7 (0.69-0.72) on this database in bootstrap resampling.
